# Lomustine with or without reirradiation for first progression of glioblastoma, LEGATO, EORTC-2227-BTG: study protocol for a randomized phase III study

**DOI:** 10.1186/s13063-024-08213-7

**Published:** 2024-06-07

**Authors:** Matthias Preusser, Tomáš Kazda, Emilie Le Rhun, Felix Sahm, Marion Smits, Jens Gempt, Johan AF Koekkoek, Angelo F Monti, Marcell Csanadi, János György Pitter, Helen Bulbek, Beatrice Fournier, Caroline Quoilin, Thierry Gorlia, Michael Weller, Giuseppe Minniti, on behalf of the European Organisation for Research, Treatment of Cancer (EORTC) Brain Tumor Group

**Affiliations:** 1 Department of Medicine I, Division of Oncology, Medical University of Vienna, Waehringer Guertel 18-20, 1090 Vienna, Austria; 2 Department of Radiation Oncology and Research Centre for Applied Molecular Oncology (RECAMO), Masaryk Memorial Cancer Institute, Brno, Czech Republic; 3 Department of Medical Oncology and Hematology, University Hospital & University of Zurich, Zurich, Switzerland; 4 Department of Neuropathology, University Hospital Heidelberg and CCU Neuropathology, DKFZ, Heidelberg, Germany; 5 Department of Radiology & Nuclear Medicine, Erasmus MC, Rotterdam, The Netherlands; 6 Department of Neurosurgery, University Medical Centre Hamburg-Eppendorf, Hamburg, Germany; 7 Department of Neurology, Leiden University Medical Centre, Leiden, The Netherlands; 8 Department of Medical Physics, ASST GOM Niguarda, Milano, Italy; 9 Syreon Research Institute, Budapest, Hungary; 10 Brainstrust—the brain cancer people, Isle of Wight, Cowes, UK; 11 EORTC Headquarters Team, Brussels, Belgium; 12 Department of Neurology, University Hospital & University of Zurich, Zurich, Switzerland; 13 Department of Radiological Sciences, Oncology and Anatomical Pathology and IRCCS Neuromed (IS), Sapienza University, Policlinico Umberto I, Rome, Italy

**Keywords:** Glioblastoma, Progression, Lomustine, Reirradiation, LEGATO, Randomized controlled trial

## Abstract

**Background:**

Chemotherapy with lomustine is widely considered as standard treatment option for progressive glioblastoma. The value of adding radiotherapy to second-line chemotherapy is not known.

**Methods:**

EORTC-2227-BTG (LEGATO, NCT05904119) is an investigator-initiated, pragmatic (PRECIS-2 score: 34 out of 45), randomized, multicenter phase III trial in patients with first progression of glioblastoma. A total of 411 patients will be randomized in a 1:1 ratio to lomustine (110 mg/m^2^ every 6 weeks) or lomustine (110 mg/m^2^ every 6weeks) plus radiotherapy (35 Gy in 10 fractions). Main eligibility criteria include histologic confirmation of glioblastoma, isocitrate dehydrogenase gene (*IDH*) wild-type per WHO 2021 classification, first progression at least 6 months after the end of prior radiotherapy, radiologically measurable disease according to RANO criteria with a maximum tumor diameter of 5 cm, and WHO performance status of 0–2. The primary efficacy endpoint is overall survival (OS) and secondary endpoints include progression-free survival, response rate, neurocognitive function, health-related quality of life, and health economic parameters. LEGATO is funded by the European Union’s Horizon Europe Research program, was activated in March 2024 and will enroll patients in 43 sites in 11 countries across Europe with study completion projected in 2028.

**Discussion:**

EORTC-2227-BTG (LEGATO) is a publicly funded pragmatic phase III trial designed to clarify the efficacy of adding reirradiation to chemotherapy with lomustine for the treatment of patients with first progression of glioblastoma.

**Trial registration:**

ClinicalTrials.gov NCT05904119. Registered before start of inclusion, 23 May 2023

**Supplementary Information:**

The online version contains supplementary material available at 10.1186/s13063-024-08213-7.

## Background

Glioblastoma is the most common primary malignant brain tumor of adults and has a fatal prognosis. Following standard first-line therapy consisting of biopsy or maximal safe neurosurgical resection followed by radiotherapy and chemotherapy with temozolomide with or without tumor-treating fields, practically all patients experience tumor progression or recurrence and further treatment lines are required [1]. Therapeutic options at second line include resection, chemotherapy, radiotherapy, experimental treatment, and best supportive care. Targeted pharmacotherapy, e.g., with specific BRAF or NTRK inhibitors, is applicable only in a minority of patients in whom druggable molecular alterations are detected [1].

Despite the fact that class I evidence is lacking, chemotherapy with lomustine is widely considered a standard treatment option at first progression of glioblastoma and has been used as comparator therapy on the control arm of several contemporary randomized clinical trials [1, 2]. Reirradiation is commonly used as a treatment option for recurrent glioblastoma in clinical routine, although there are very limited prospective data [3–5]. A number of small studies documented a favorable safety profile for reirradiation delivered in different regimens and with or without various chemotherapies [3, 4, 6–13]. High level of evidence data on efficacy and impact on the neurological status and quality of life are lacking.

EORTC-2227-BTG (LEGATO, NCT05904119) was designed as pragmatic clinical trial (PRECIS-2 score: 34 out of 45 as defined by EORTC headquarters using http://precis-2.org, Table 1) [14] to test the hypothesis that the addition of radiotherapy to lomustine chemotherapy is beneficial for the treatment of progressive glioblastoma. This trial is funded by the European Union’s Horizon Europe Research program (project number: 101103655, 2227@eortc.org). This is an investigator-initiated clinical trial. Therefore, the funders played no role in the design of the study and collection, analysis, and interpretation of data and in writing the manuscript. Here, we summarize the clinical trial design of this ongoing study with first site activation achieved in March 2024 and a projected study completion by 2028.

**Table 1:** PRECIS-2 scoring of the LEGATO trial. *1* very explanatory, *2* rather explanatory, *3* equally explanatory and pragmatic, *4* rather pragmatic, and *5* very pragmatic

PRECIS-2 domain	Rating
Eligibility	2
Recruitment	4
Setting	5
Organization	3
Fexibility in treatment delivery	4
Flexibility in adherence	4
Follow-up	2
Primary outcome	5
Primary analysis	5
Total	34

## Study design

**Fig. 1:**
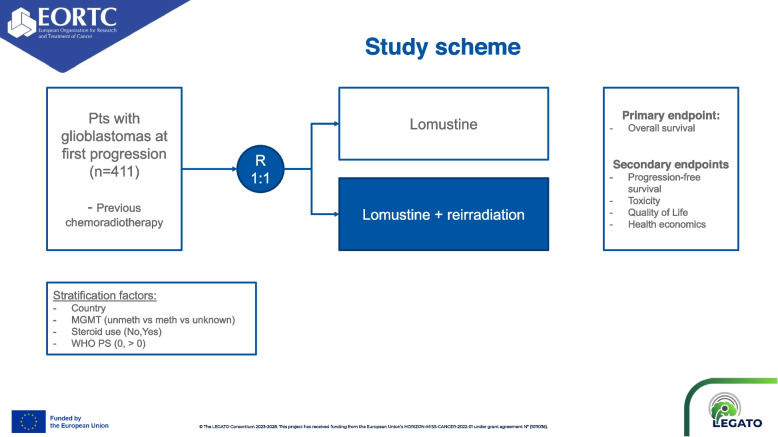
Study scheme of the EORTC-2227-BTG (LEGATO) trial

EORTC-2227-BTG (LEGATO, NCT05904119) is an investigator-initiated, pragmatic, randomized, open-label, multicenter phase III trial in patients with first progression of glioblastoma. Figure 1 summarizes the study design. Of note, the information provided reflects the protocol version 3.0 approved on 24 January 2024. Investigators must refer to the latest version of the full study protocol, which is available to study investigators in their trial files. Publicly available information and updates on the trial progress can be found at https://legato-horizon.eu.

### Trial population

Main eligibility criteria include age ≥18 years, written informed consent, histologic confirmation of glioblastoma, IDH wild-type per WHO 2021 classification, first progression or recurrence after first-line treatment with biopsy or maximal safe resection and standard radiotherapy or chemoradiotherapy having occurred at least 6 months after the end of prior radiotherapy, radiologically measurable disease according to RANO criteria with a maximum tumor diameter of 5 cm, WHO performance status of 0–2, and any *MGMT* promoter methylation status. The detailed eligibility criteria are shown in Table 2. The patients will receive extensive information about the study set-up and requirements during the recruitment by the local investigators, who are board-certified physicians with current good clinical practice (GCP) certification and confirmed delegation and completed training for clinical trial activities. The importance of completion of the follow-up will be stressed. A clinical trial insurance has been taken out according to the laws of the countries where the study will be conducted.

**Table 2:** Eligibility criteria for the EORTC-2227-BTG (LEGATO) trial.

Inclusion criteria	Comment	Exclusion criteria
Before patient’s enrolment, written informed consent must be given according to ICH/GCP, and national/local regulations.		Any prior anticancer treatment for recurrent glioblastoma (except surgery).
Patients with first progression or recurrent glioblastoma after first-line treatment with biopsy or maximal safe resection and standard radiotherapy or chemoradiotherapy having occurred at least 6 months after the end of prior radiotherapy.	Prior first-line therapy may include any systemic antineoplastic treatment other than nitroureas, tumor-treating fields, conventionally fractionated or abbreviated (minimum 15 fractions) radiotherapy.	Significant reduction in thrombocyte and/or leukocyte counts (leukocytes < 4000/mm*3* and/or the platelets < 100,000/mm3) as well as severe renal impairment according to investigator's opinion.
Measurable disease according to RANO criteria with a maximum tumor diameter of 5 cm (local investigator assessment).	In case of multiple lesions, maximum cumulative CTV diameter of 5 cm treatable by one isocenter.	Previous (last dose in the 15 days prior to lomustine initiation) or ongoing salicylates due to increased risk of bleeding in case of thrombocytopenia.
Candidates for treatment with lomustine as per physician’s assessment.		History or present acute leukemia or any myeloid disease.
In case of surgery for recurrence: fully recovered from surgery, confirmation of recurrence by histology, and patient fit for treatment as per local investigator assessment.		Known hypersensitivity to the active components or excipients of lomustine.
Histologically proven diagnosis of glioblastoma, IDH wild-type per WHO 2021 classification and local assessment of tissue from diagnosis or recurrence.		Concurrent or recent history (30 days prior to lomustine initiation) of varicella (infection or exposure) and herpes zoster.
Stable or decreasing dose of steroids for 7 days prior to enrolment		Known hereditary galactose intolerance, Lapp-lactase deficiency, or glucose-galactose malabsorption.
Age ≥ 18 years		Known coeliac disease or wheat allergy.
WHO performance status of 0–2		Patients with pulmonary infiltration, interstitial pneumonia, or pulmonary fibrosis and with a baseline below 70% of the predicted FVC or DLCO.
Women of childbearing potential must have a negative serum pregnancy test within 7 days prior to the first dose of study treatment.		Live attenuated vaccine in the 3 months prior to lomustine initiation.
Patients of childbearing/reproductive potential must agree to use adequate birth control measures during the study treatment period and for at least 6 months after the last dose of study treatment. A highly effective method of birth control is defined as a method which results in a low failure rate (i.e., less than 1% per year) when used consistently and correctly.		Any serious or uncontrolled medical condition (e.g., infections, chronic alcoholism, drug addiction) or abnormality, in the judgment of the investigator that prohibits obtaining informed consent, safe participation, and study completion.
Female subjects who are breastfeeding should discontinue nursing prior to the first dose of study treatment and until 6 months after the last study treatment.		Known contraindication to imaging tracer or any product of contrast media and MRI contraindications.
Non-sterile males must use contraception during treatment and for 6 months after the last dose.		Any psychological, familial, sociological, or geographical condition potentially hampering compliance with the study protocol and follow-up schedule; those conditions should be assessed and discussed with the patient before the enrolment in the trial.
Non-sterile males must avoid sperm donation for the duration of the study and for at least 6 months after the last dose of study treatment.		

*DLCO* Carbon monoxide diffusing capacity, *FVC* Forced vital capacity, *GCP* Good clinical practice, *ICH* International Council of Harmonization, *IDH* Isocitrate dehydrogenase gene, *MRI* Magnetic resonance imaging, *RANO* Response Assessment in Neuro-Oncology, *WHO* World Health Organization

### Objectives and endpoints

The objectives and endpoints of the LEGATO trial are detailed in Table 3. In brief, the primary efficacy endpoint is overall survival (OS) and secondary endpoints include progression-free survival (PFS), response rate, neurocognitive function, health-related quality of life, and health economic parameters.

**Table 3:** Objectives and endpoints of the EORTC-2227-BTG (LEGATO) trial

**Objectives**	**Endpoints**
**Primary objective**	**Primary endpoint**
To show that lomustine and radiotherapy improves overall survival in the study population as compared to standard treatment.	Overall survival from the date of enrolment.
**Secondary objectives**	**Secondary endpoint**
To show that lomustine and radiotherapy improves progression-free survival in the study population as compared to standard treatment.	Progression-free survival from enrolment per RANO criteria as assessed by the local investigator.
To show that lomustine and radiotherapy improves response in the study population as compared to standard treatment.	Objective response per RANO criteria as assessed by the local investigator.
To assess the toxicity profile of lomustine plus reirradiation.	Safety according to the CTCAE 5.0.
To assess whether lomustine plus reirradiation improves QDFS as compared to lomustine alone. To assess the difference in secondary and exploratory QoL scales between lomustine plus reirradiation and lomustine alone.	QDFS survival defined as a deterioration event of ≥10-point worsening from baseline in the GHQ without further improvement (i.e., no subsequent ≥ 10-point improvement) or death due to any cause while on treatment
To assess patient’s neurocognitive functioning with lomustine plus reirradiation.	Patient’s neurocognitive functioning assessed by MMSE.
To assess health economics with lomustine plus reirradiation.	Health utility values

*CTCAE* Common Terminology Criteria of Adverse Events, *GHQ* General health questionnaire, *MMSE* Mini mental state exam, *QDFS* Quality of live deterioration free survival, *QoL* Quality of life, *RANO* Response Assessment in Neuro-Oncology

OS is defined as the number of days from the date of enrolment to the date of death due to any cause. If a subject has not died, the data will be censored at the last date documented to be alive. PFS will be defined as the number of days from the date of enrolment to the date of earliest radiological disease progression or to the date of death due to any cause, if disease progression did not occur. Patients for whom neither death nor progression have been documented will be censored at the date of the last radiological assessment that the patient was progression-free.

Radiological follow-up for the evaluation of PFS and response rate will be performed by cranial MRI every 12 weeks. MRI will be assessed according to Response Assessment in Neuro Oncology (RANO) criteria interpretation by the local investigator.

Toxicity reporting will be done according to the Common Terminology Criteria for Adverse Events (CTCAE) version 5.0. Neurocognitive functioning will be assessed using Mini-Mental State Examination (MMSE) every 6 weeks and the NANO scale will be utilized for evaluation of neurological function. Health-related Quality of Life (HRQoL) will be assessed every 6 weeks using EORTC Quality of Life Questionnaire (QLQ)-C30, QLQ-BN20 Brain tumor module and one additional item from the EORTC Item Library (IL)-46.

Self-reported HRQoL data will be transformed into health utility values for subsequent health economic analyses. To this end, the EORTC QLQ-C30 data collected for each study subject and at each HRQoL assessment time point will be mapped to health utility values using an established indirect mapping approach [15, 16]. In the adopted indirect mapping approach, first the probabilities of belonging to European Quality-of-Life-5 Dimensions (EQ-5D) categories will be calculated, and then the corresponding EQ-5D scores will be translated into health utility values using a country-specific value set for the conversion [16].

Tables 4, 5, and 6 summarize study calendar before treatment start, during treatment, and during follow-up. Data will be captured in electronic case report forms.

**Table 4:** Study calendar—before treatment started

Assessment	Within 14 days prior to enrolment	Within 7 days prior to enrolment
Disease evaluation (cMRI)	X	
Complete medical history	X	
Concomitant medication		X
Vital signs		X
ECOG/WHO performance status		X
Clinical examination		X
Pulmonary function test		X
Mini-Mental State Examination (MMSE)		X
NANO scale		X
12-lead ECG		X
Hematology		X
Serum chemistry		X
Coagulation		X
Pregnancy test for WOCBP		X
Health-related quality of life (HRQoL)		X
Elderly Minimal Dataset Comprehensive Geriatric Assessment (G8) for patients ≥ 70 years of age		X

*cMRI* contrast magnetic resonance imaging, *ECOG/WHO* Eastern Cooperative Oncology Group/World Health organization, *MMSE* Mini Mental State Examination, *NANO* Neurologic Assessment in Neuro-Oncology, *ECG* Electrocardiogram, *WOCBP* Women of childbearing potential, *HRQoL* Health-related quality of life

**Table 5:** Study calendar—during protocol treatment

Assessment	End of RT	Every 6 weeks	Days 28 and 35 of each 6-week cycle	Every 12 weeks	End of study treatment
	Experimental arm only	Both treatment arms	Both treatment arms	Both treatment arms	30 (± 7 days) after last dose
Disease evaluation (cMRI)				X	
Assessments of adverse events	X	X			X
Concomitant medication		X			X
Vital signs		X			X
ECOG/WHO performance status	X	X			X
Clinical examination		X			X
Pulmonary function test		X			X
Mini-Mental State Examination (MMSE)		X			X
NANO scale		X (optional)			X (mandatory)
12-lead ECG					X
Hematology		X	X		X
Serum chemistry		X			X
Coagulation		X			X
Pregnancy test for WOCBP		X			X
Health-related Quality of Life (HRQoL)		X			X

*RT* radiotherapy, *cMRI* contrast magnetic resonance imaging, *ECOG/WHO* Eastern Cooperative Oncology Group/World Health organization, *MMSE* Mini Mental State Examination, *NANO* Neurologic Assessment in Neuro-Oncology, *ECG* electrocardiogram, *WOCBP* women of childbearing potential, *HRQoL* health-related quality of life

**Table 6:** Study calendar—follow-up

**Assessment**	**Absence of disease progression**	**Disease progression**
	**Every 12 weeks ± 14 days after last treatment administration**	
Survival status	X	X
Diagnosis of new malignancy	X	X
Subsequent anti-cancer therapy	X	X
Pregnancy test for WOCBP	Pregnancy test is to be renewed/repeated for 6 months after last protocol treatment	
Health-related quality of life (HRQoL)	X	X
Disease evaluation (cMRI)	X	

*WOCBP* Women of childbearing potential, *HRQoL* Health-related quality of life, *cMRI* Contrast magnetic resonance imaging

### Statistical considerations and data management

A total sample size of 411 patients is needed to detect an increase from an expected median OS of 9 months [17, 18] in the control arm to 12.5 months in the experimental arm based on a one-sided log-rank test at a significance level of 2.5% and a power of 80%, corresponding to a hazard ratio of 0.72. An increase of 3.5 months of survival when radiotherapy is added to lomustine would be considered clinically significant. Stratification factors will include country, *MGMT* promoter methylation status (unmethylated vs methylated vs unknown), steroid use at study entry (no, yes), and WHO performance status (0, > 0). There will be an interim futility and efficacy analysis organized after observation of 97 OS events (33%). The primary analysis will be performed in the intent-to-treat population (ITT), i.e., all randomized patients according to the arm they were allocated to. In the per protocol population, all randomized patients who have started their allocated treatment (i.e., at least one dose of lomustine or lomustine and radiotherapy) will be analyzed. In the per protocol population, patients will be classified and analyzed in the arm they were assigned at the time of enrolment. For data management procedures, EORTC, in its role of sponsor and data controller of the clinical study ensures that the processing activities on the personal data in scope of this study are compliant with, but not limited to, the requirements set by EU General Data Protection Regulation (GDPR EU 2016/679), its subsequent amendments and any additional national laws, recommendations, and guidelines as applicable. All data are collected via an electronic case report form by study staff with confirmed GCP certification named on delegation logs at the trial sites and are stored in secure database at EORTC Headquarter. The name of patients enrolled in the trial will neither be asked for nor recorded at the EORTC headquarters. A sequential identification number will be automatically allocated to each patient registered in the trial. This number will identify the patient and will be included on all case report forms and corresponding material and data associated with the patient. In order to avoid identification errors, the patient’s code (maximum of four alpha numerics) and year of birth will also be reported on the case report forms. Data collected during the course of the research will be kept strictly confidential and only accessed by members of the trial team (or individuals from the Sponsor organization or center sites where relevant to the trial). The EORTC headquarters will perform on-site and/or remote monitoring visits according to the approved study monitoring plan in order to maximize protocol adherence, correct false data entries, and minimize missing data. No imputation will be used to account for missing data. The first visit in a participating site will be performed within 6 to 12 months after the first patient’s enrolment at this site. Frequency and number of subsequent visits will depend on site’s accrual and quality observed during the previous visit. Reporting of adverse events indicating expectedness, seriousness, severity, and causality will be performed according to ICH GCP and EU Regulation 536/2014. At the trial sites, all reporting of adverse events must be done by the principal investigator or authorized staff member and will be transmitted electronically to the pharmacovigilance department of EORTC. As the sponsor, EORTC will be responsible for the reporting of suspected unexpected serious adverse reaction (SUSARs)/unexpected serious adverse reaction (SARs) to the competent authorities, ethics committees, EudraVigilance Clinical Trial Module (EVCTM), and all participating investigators as applicable. Medical review will be performed on a regular basis by a medical representative at EORTC and with the support of the principal investigator. Data collected during the course of the research will be kept strictly confidential and only accessed by members of the trial team (or individuals from the sponsor organization or center sites where relevant to the trial). The independent data monitoring committee for EORTC studies (IDMC) is in charge of the independent oversight of this study, according to the EORTC policies. LEGATO is an open-label trial and trial participants, care providers, outcome assessors, and data analysts will not be blinded. The clinical trial is registered at ClinicalTrials.gov with the identification number NCT05904119.

### Interventions

Patients will be randomized by EORTC in a 1:1 ratio to the control arm of lomustine alone or the experimental arm of lomustine plus radiotherapy.

#### Control arm: lomustine

In the control arm, lomustine will be given at a recommended dose of 110 mg/m^2^ (maximum absolute dose 200 mg, minimum dose 80 mg) every 6 weeks. The maximum cumulative dose must not exceed 1000 mg/m^2^ to prevent pulmonary toxicity [19]. Participants must start lomustine within 7 days of enrolment. Treatment will be administered until disease progression, unacceptable toxicity, death, or until the occurrence of any predefined withdrawal criterion such as withdrawal of patient consent or safety concerns. Lomustine has a well-known safety profile that includes fatigue, hematological symptoms, and on rare occasions, the development of pulmonary toxicity after more than 6 months of treatment and a cumulative dose of 1000 mg/m^2^. Hematological toxicity, as the main concern in the treatment by lomustine occurs usually after 4 weeks after drug administration. Recommendations for dose modifications follow published guidelines [20].

#### Experimental arm: lomustine plus reirradiation

In the experimental arm, radiotherapy with a prescribed dose of 35 Gy (daily dose 3.5 Gy, 10 fractions) over 2 weeks will be delivered in addition to the same chemotherapy regimen as in the control arm (lomustine 110 mg/m^2^ every 6 weeks). Lomustine should be started within 7 days of enrolment. Radiotherapy should start within 14 days of randomization and/or within 7 days of the first lomustine intake. Megavoltage equipment able to deliver stereotactic, intensity-modulated radiotherapy (IMRT) or volumetric modulated arc therapy (VMAT) is required.

Radiotherapy delineation will be performed on a magnetic resonance (MR) image with contrast agent administration. Treatment planning using volumetric modulated arc therapy (VMAT) and image-guided radiation therapy (IGRT) is advised. IMRT or VMAT planning is allowed. Intensity-modulated radiation therapy (IMRT) and three-dimensional conformal radiotherapy are allowed.

The maximum diameter of contrast enhanced recurrent tumor allowed for inclusion in the trial is 5 cm. In case of multifocal disease, the lesions must be in proximity to one another, to be treated with a single isocenter, and the maximum cumulative tumor diameter is 5 cm.

The gross tumor volume (GTV) will be defined using the MRI images as a T1-weighted contrast enhancing lesion (contrast-enhanced CT for patients who cannot undergo MRI). In patients who undergo surgery, the GTV is defined by the post-operative resection cavity plus any residual enhancing tumor.

A clinical tumor volume (CTV) expansion of maximum 5 mm can be applied at the investigator discretion for lesions measuring less than 4 cm in maximum diameter or for new lesions. CTV is cropped out around natural fixed barriers for tumor spread (skull, falx, tentorium). Otherwise, no additional CTV expansion will be added.

An appropriate planning target volume (PTV) expansion, justified based on image guidance and immobilization, will be applied. Regardless, the PTV expansion should be no smaller than 2 mm. Daily image-guided radiation therapy (IGRT) is required for institutions utilizing PTV margins of less than 5 mm.

Normal tissue limits will be defined according to the ESTRO-EORTC consensus on reirradiation, reflecting possible overlap with previous radiation target or critical organs at risk with concern of toxicity from cumulative doses [21].

The main toxicity expected from re-irradiation is radionecrosis in less than 10% of cases. The increased, synergistic, risk of hematological toxicity in the combination of lomustine and reirradiation is expected to be minimal.

### Study intervention compliance

A record of the number of tablets dispensed to and taken by each patient will be maintained and reconciled with study intervention and compliance records. Intervention start and stop dates, including dates for intervention delays and/or dose reductions will also be recorded in the electronic case report forms.

### Supportive care and concomitant medications

Supportive care is left at the investigator’s discretion, but adherence to the EANO recommendations is advised [20].

Prohibited medications include live or attenuated vaccines at any time during the study and for a period of 3 months after treatment discontinuation. Yellow fever vaccine is strictly contraindicated because of the risk of fatal systemic vaccinal disease. Patients must not receive salicylates due to increased risk of bleeding in case of thrombocytopenia. Any concomitant systemic therapy intended for the treatment of cancer, whether health authority-approved or experimental, is prohibited.

Permitted medications considered necessary for a participant’s welfare according to the local investigator include growth factors in accordance with ASCO guidelines for secondary prophylaxis and steroids as anti-emetics or part of symptom management [22].

SPIRIT reporting guidelines for publication of clinical trials protocols [23] were used and were submitted as an additional file (see Additional file 1).

### Study governance and trial sites

The EORTC is the legal sponsor of the LEGATO trial. The Study Management Group (SMG) consists of the EORTC Headquarters team in charge of running the study (clinical research physician, statistician, clinical scientist, project manager, and data managers) and the principal study coordinators. The EORTC headquarter team is responsible for the day-to-day conduct of the trial. The study coordinator will assist the team in case of problems with patient evaluation (eligibility, treatment compliance, safety). The SMG also performs the medical review. The Study Steering Committee (SSC) for this study is composed at of the study coordinators and two representatives (clinical scientist and statistician) of the EORTC headquarters (study clinical research physician or clinical scientist or statistician). This committee provides the general oversight of the study and has executive power. The SSC monitors study progress and conduct and advises on its scientific credibility. The SSC will consider and act, as appropriate, upon the recommendations of the Independent Data Monitoring Committee. There is a patient representative in the protocol writing committee, which is composed of a total of 13 persons from various disciplines. LEGATO was activated in March 2024 and will enroll patients in 43 sites in 11 countries across Europe including Austria, Belgium, Czech Republic, Denmark, France, Germany, Italy, Norway, Spain, Switzerland, and The Netherlands. Information on the LEGATO trial network can be found at https://www.clinicaltrials.gov/study/NCT05904119 and https://legato-horizon.eu.

## Discussion

LEGATO is the first randomized phase III trial investigating the efficacy of adding irradiation to lomustine chemotherapy at first progression of glioblastoma and is designed to answer a clinically relevant question being regularly discussed in patient consultations and multidisciplinary tumor boards all over the world. The eligibility criteria, study-related procedures, and outcomes are kept to a minimum in order to deliver a pragmatic and efficient clinical trial with high relevance to physicians, patients, policy makers, and other stakeholders. The clinical trial design follows standard state-of-the-art conventions and defines OS as primary efficacy endpoint and PFS, response rate, neurocognitive function, and HRQoL as secondary endpoints. In order to increase the relevance of the data for application in different health care settings, LEGATO is also investigating health economic parameters.

In conclusion, LEGATO is the first prospective randomized clinical phase III trial comparing lomustine chemotherapy with the combination of lomustine and irradiation for the therapy of progressive glioblastoma and will provide robust data that will guide everyday practice in clinical neuro-oncology.

## Trial status

Protocol version 3.0 was approved on 24 January 2024. All amendments will be notified to the sites and to all competent authorities. 3 April 2024 is the date the recruitment began. The approximate date when recruitment will be completed is Q4 2026.

### Supplementary Information

Supplementary Material 1. 

## Data Availability

The datasets used and/or analyzed during the study will be made available according to EORTC and European Union’s HORIZON-MISS-CANCER-2022-01 policy. Plans for investigators and sponsor to communicate trial results to participants, healthcare professionals, the public, and other relevant groups include presentations at scientific meetings, publications in peer-reviewed scientific journals, and information of the public via a dedicated homepage, a newsletter, and social media postings (https://legato-horizon.eu). Storage of biological specimens for genetic or molecular analysis are not foreseen in the current trial. EORTC is committed to ensuring that the data generated from its studies be put to good use by the cancer research community and, whenever possible, are translated to deliver patient benefit. It is therefore EORTC’s policy to consider for sharing upon request from qualified scientific and medical researchers all data generated from its research while safeguarding intellectual property, the privacy of patients, and confidentiality. Requests for accessing the data of published trials should be filed through the data-sharing tab on EORTC website (www.eortc.org).

## Abbreviations

BTG: Brain tumor group
EORTC: European Organisation for Research and Treatment of Cancer
PRECIS: Pragmatic explanatory continuum indicator summary
IDH: Isocitrate dehydrogenase gene
WHO: World Health Organization
RANO: Response Assessment in Neuro Oncology
OS: Overall survival
BRAF: v-Raf murine sarcoma viral oncogene homolog B
NTRK: Neurotrophic tyrosine receptor kinase
MGMT: O6-methylguanine DNA methyltransferase
PFS: Progression-free survival
MRI: Magnetic resonance imaging
CTCAE: Common Terminology Criteria for Adverse Events
MMSE: Mini Mental State Examination
NANO: Neurologic Assessment in Neuro-Oncology
HRQoL: Health-related quality of life
QLQ-C: Quality of Life Questionnaire-Cancer
QLQ-BN: Quality of Life Questionnaire-Brain
EQ-5D: European Quality-of-Life-5 Dimensions
ITT: Intent-to-treat population
IDMC: Independent data monitoring committee
IMRT: Intensity-modulated radiotherapy
VMAT: Volumetric modulated arc therapy
IGRT: Image-guided radiation therapy
GTV: Gross tumor volume
CT: Computed tomography
CTV: Clinical tumor volume
PTV: Planning target volume
ESTRO: European Society for Radiotherapy and Oncology
EANO: European Association of Neuro-Oncology
ASCO: American Society for Clinical Oncology
SPIRIT: Standard Protocol Items: Recommendations for Interventional Trials
ICH/GCP: International Council of Harmonization/Good Clinical Practice
DLCO: Carbon monoxide diffusing capacity
FVC: Forced vital capacity
GHQ: General health questionnaire
cMRI: Contrast magnetic resonance imaging
ECOG: Eastern Cooperative Oncology Group
ECG: Electrocardiogram
WOCBP: Women of childbearing potential
